# Micro-RNA30c Negatively Regulates REDD1 Expression in Human Hematopoietic and Osteoblast Cells after Gamma-Irradiation

**DOI:** 10.1371/journal.pone.0048700

**Published:** 2012-11-07

**Authors:** Xiang Hong Li, Cam T. Ha, Dadin Fu, Mang Xiao

**Affiliations:** Radiation Countermeasures Program, Armed Forces Radiobiology Research Institute, Uniformed Services University of the Health Sciences, Bethesda, Maryland, United States of America; Rush University Medical Center, United States of America

## Abstract

We recently demonstrated that a novel cell stress response gene REDD1 protects human fetal osteoblast cell line (hFOB) cells from γ-radiation-induced premature senescence. Here we show that levels of endogenous REDD1 are very low in human hematopoietic progenitor CD34+ cells regardless of radiation, but highly expressed in differentiated hematopoietic cells (14 day cultured CD34+ cells) in response to radiation, which might be associated with radiation tolerance of the latter cells. To further understand the mechanisms of radiation-induced damage in different cells, microRNA (miRNA)-arrays were performed using purified miRNAs from CD34+ and hFOB cells before and post-irradiation and real-time reverse transcription (RT)-PCR was used to validate the expression profiles of miRNAs in the radiation-damaged cells. The results indicate that γ-radiation downregulated 16 miRNAs in CD34+ cells and 14 in hFOB cells. Radiation-induced upregulation was observed for 15 miRNAs in CD34+ cells and 18 miRNAs in hFOB cells. The profiles of radiation-induced miRNA expression were completely different in CD34+ vs. hFOB cells. Radiation up-regulated miRNA (miR)-30b, miR-30c and miR-30d in CD34+ cells, whereas it inhibited miR-30c expression in hFOB cells. Since miR-30 has potential target sites located in the 3′untranslated region (UTR) of the REDD1 gene and radiation regulated miR-30c expression in both CD34+ and hFOB cells, we further explored the effects of miR-30c on REDD1 expression using miR-30c inhibitor and precursor (pre-miR-30c). The results show that pre-miR-30c transfection suppressed REDD1 expression in 14 day cultured CD34+ cells and hFOB cells and resulted in hFOB cell death. In contrast, inhibition of miR-30c expression significantly enhanced clonogenicity in CD34+ cells. Our data suggest that CD34+ and hFOB cells have different miRNA expression patterns after irradiation and miR-30c plays a key role in radiation-induced cell damage which might be through regulation of REDD1 expression.

## Introduction

Radiotherapy is commonly used for cancer treatment. However, it often results in side effects due to radiation damage in normal tissue [1], [2]. Bone marrow (BM) toxicity is the dose-limiting factor for radiotherapy and radioimmunotherapy in cancer patients. Adult hematopoietic stem and progenitor cells (HSPC) reside in BM next to the endosteal bone surface, which is lined primarily by hematopoietic niche osteoblastic cells. Survival of bone marrow osteoblasts is critical for the restoration of hematopoiesis after radioablation. We have demonstrated that the γ-radiation responsive features of HSPC and hematopoietic niche osteoblast cells are different because radiation caused death of primary human hematopoietic CD34+ cells through apoptosis [3], whereas it induced senescence in human fetal osteoblast cell line (hFOB) cells [4]. However, osteoblasts are relatively more radiation-resistant than HSPCs. The mechanisms leading different radiation responses in HSPC and osteoblasts have not been elucidated. To further understand the mechanisms of radiation-induced damage in different cells, in the present study microRNA (miRNA) arrays were performed using purified miRNAs from CD34+ and hFOB cells before and post-γ-irradiation. Real-time reverse transcription (RT)-PCR was used to validate expression profiles of miRNAs in the radiation-damaged cells.

miRNAs are short ribonucleic acid (RNA) molecules (on average only 22 nucleotides long) found in eukaryotic cells and belong to the single-stranded small non-coding RNA family [5], [6]. miRNAs are post-transcriptional regulators that bind to the 3′untranslated region (UTR) of specific target messenger RNA transcripts (mRNAs), usually resulting in translational repression or target degradation and gene silencing. miRNA-mediated gene repression occurs through both translational repression and mRNA destabilization [7], [8]. Mammalian genomes encode hundreds of conserved miRNAs, which target mammalian genes and are abundant in many human cell types. miRNAs could regulate the cellular changes required to establish the stress-induced cell damage phenotype [9]. In the present study, we found that the expression profiles of miRNA in human hematopoietic progenitor CD34+ cells and osteoblast cells after γ-irradiation are completely different. Furthermore, our data show that radiation regulates miR-30 expression in the opposite manner in CD34+ and hFOB cells, with enhanced miR-30b, miR-30c and miR-30d expression in CD34+ cells (which are sensitive to radiation damage), and decreased miR-30c expression in the relatively radio-resistant hFOB cells. Recent studies suggested that miR-30 is one of the most common known tumor suppressor miRNAs [10]. miR-30 family members are involved in regulation of p53-induced mitochondrial fission and cell apoptosis [11], regulation of B-Myb expression during cellular senescence [12], and play important roles in epithelial, mesenchymal, osteoblast cell growth and differentiation [13]-[15]. We recently reported that a novel cell stress response gene REDD1 [16], [17] was highly induced in hFOB cells and protected these cells from radiation-induced damage. Knockdown of REDD1 by siRNA resulted in hFOB cell number decreases. In contrast, over-expression of REDD1 inhibited mTOR and p21 expression, suppressed inflammatory factor secretion and protected these cells from γ-radiation-induced senescence. Interestingly, miR-30 has potential target sites located in the 3′UTR of REDD1 gene, and we show here that REDD1 is a target of miR30c in response to γ-radiation in primary human hematopoietic CD34+ and hFOB cells. Hence manipulation of miR-30 may be a useful approach to explore the mechanisms of radiation-induced apoptosis and/or premature senescence in mammalian hematopoietic tissues.

## Results

### miRNA Microarray

**Figure 1:**
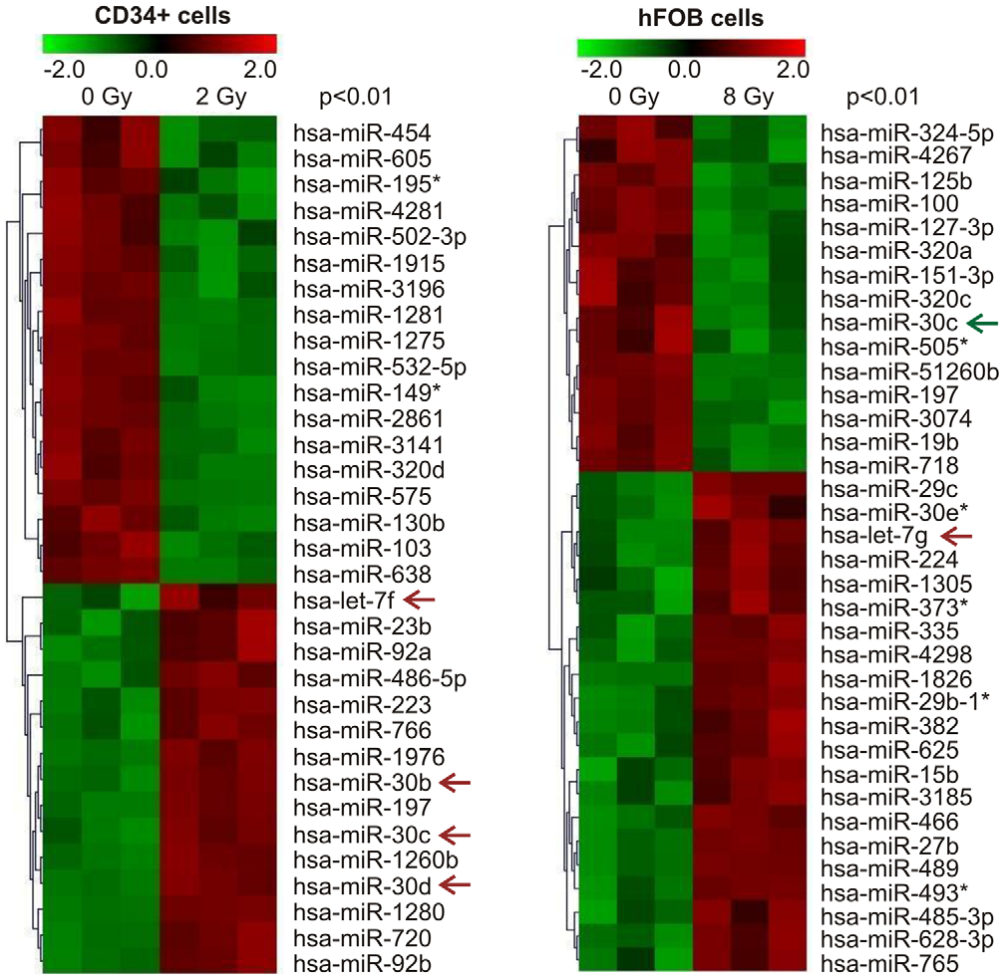
Differentially expressed miRNAs in human hematopoietic and osteoblast cells following exposure to γ- radiation. Human hematopoietic CD34+ cells and fetal osteoblastic cell line (hFOB) cells were exposed to 2 Gy or 8 Gy of γ- radiation, respectively. miRNA microarray analysis was performed by LC Sciences Co. (Houston, Texas) in triplicate. Significant increases or decreases in miRNA expression in irradiated vs. non-irradiated control samples were strictly cut off at p<0.01. The three columns represent three biological replicates from each sample. Colors represent log2 values from −2.0 to +2.0. Arrows indicate miRNAs that either increased (red) or decreased (green) after radiation. *: miRNA expressed at low levels.

To determine miRNA expression in HSPC and hematopoietic niche osteoblasts after ionizing radiation (IR), human CD34+ cells and hFOB cells were exposed to 2 or 8 Gy γ-radiation that had been previously determined to generate one and two logs of cell kill by clonogenic assay, respectively [3], [18]. One h after exposure, cells were collected and miRNA were purified as described in Materials and Methods. miRNA microarray analysis in triplicate was performed by LC Sciences Co. (Houston, Texas) to probe for all known human miRNA species. Radiation-induced increases or decreases in miRNA expression are shown for CD34+ and hFOB cells in Figure 1 for changes where p<0.01. Analysis revealed that γ-radiation altered the expression of 31 miRNA species (16 downregulated and 15 upregulated) in CD34+ cells and 32 miRNA species (14 downregulated and 18 upregulated) in hFOB cells. The profiles of miRNA expression in human CD34+ cells and osteoblast cells in response to γ-radiation were completely different, and only Let-7 and miR-30 miRNA families were regulated by radiation in both types of cells (Table 1) with increased Let-7f in CD34+ cells and increased let-7g in hFOB cells. Interestingly, miR-30 family member expression in irradiated CD34+ and hFOB cells were changed in opposite directions. miR-30b, miR-30c and miR-30d were upregulated in CD34+ cells whereas miR-30c was downregulated in hFOB cells. We asked whether the different features of miR-30 in these cells were associated with their radiation sensitivity and decided to evaluate the effects of miR-30 on radiation-injured CD34+ and hFOB cells.

**Table 1:** Radiation regulated miRNAs expression in both CD34+ and hFOB cells.

CD34+ cells		hFOB cells	
Ratio:	2 Gy/0 Gy (Log 2)		8 Gy/0 Gy (Log 2)
hsa-let-7f	0.19	hsa-let-7g	0.44
hsa-miR-30b	0.88	hsa-miR-30c	−0.24
hsa-miR-30c	0.79		
hsa-miR-30d	0.56		
	P<0.01		P<0.01

Data from miRNA Microarray (LC Sciences Co. Houston, Texas).

### miRNA Real-Time RT-PCR validation of miR-30 family member expressions in CD34+ and hFOB cells

**Figure 2:**
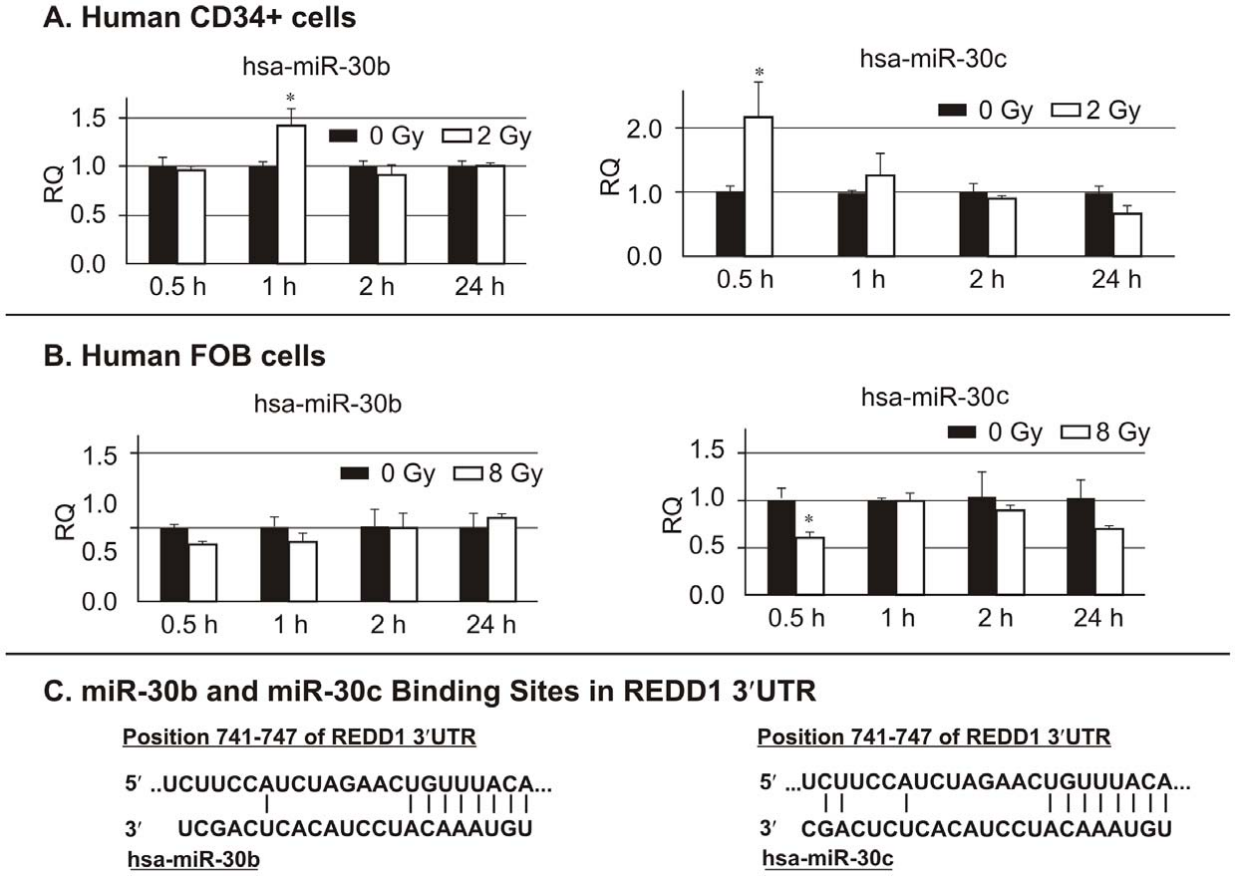
hsa-miR-30 expression after γ-irradiation (Quantitative Real Time-PCR). CD34+and hFOB cells were collected at 0.5, 1, 2 and 24 h after exposure to 2 or 8 Gy radiation, respectively. miR-30b and miR30c expression were evaluated by RT-PCR. U6 was used as a loading control. miR-30b and miR-30c levels were increased at 0.5 or 1 h after γ-irradiation in (A) CD34+ cells, whereas they were decreased or not altered in (B) hFOB cells in response to radiation. Results represent one of 3 independent experiments. Means ± SD. *, p<0.05. (C) miR-30b and miR-30c binding sites in REDD1 3′UTR are shown. RQ: relative quantification.

To confirm the miRNA microarray findings, the changes in miRNA expression for miR-30 family were validated by RT-PCR. miR-30 family members, miR -30a,-30b, -30c, 30d and 30e, were examined in both irradiated and non-irradiated CD34+ and hFOB cells using quantitative real-time RT-PCR. Consistent with miRNA microarray data, RT-PCR results demonstrated that 2 Gy γ-radiation significantly induced miR-30b and miR-30c expression in CD34+ cells. miR-30b was increased by 0.4 fold 1 h after irradiation and miR30c was increased by 1.2 fold 0.5 h after irradiation compared to non-irradiated control CD34+ cells (Figure 2A). At 2 h after irradiation, levels of miR-30b and miR-30c returned to baseline as shown in non-irradiated cells and did not change thereafter. In contrast with CD34+ cells, levels of miR-30b did not change and miR-30c was reduced 0.4 fold in hFOB cells by 8 Gy γ-irradiation (Figure 2B). miR-30a, miR-30d and miR-30e expression was not significantly altered after γ-radiation in CD34+ and hFOB cells as revealed by RT-PCR (data not shown). Next, we analyzed potential targets of miR-30 family members using the miRNA target prediction database TargetScan 5.1 (http://www.targetscan.org), and found that members of the miR-30 family were predicted to target the stress-response gene REDD1. Figure 2C shows the miR-30b and miR-30c binding site in the 3′UTR of the *REDD1* gene. Hence we explored interactions between the miR-30 family and REDD1.

### REDD1 expression was higher in differentiated hematopoietic cells and these cells were more resistant to γ-radiation than their precursor, hematopoietic progenitor cells

**Figure 3:**
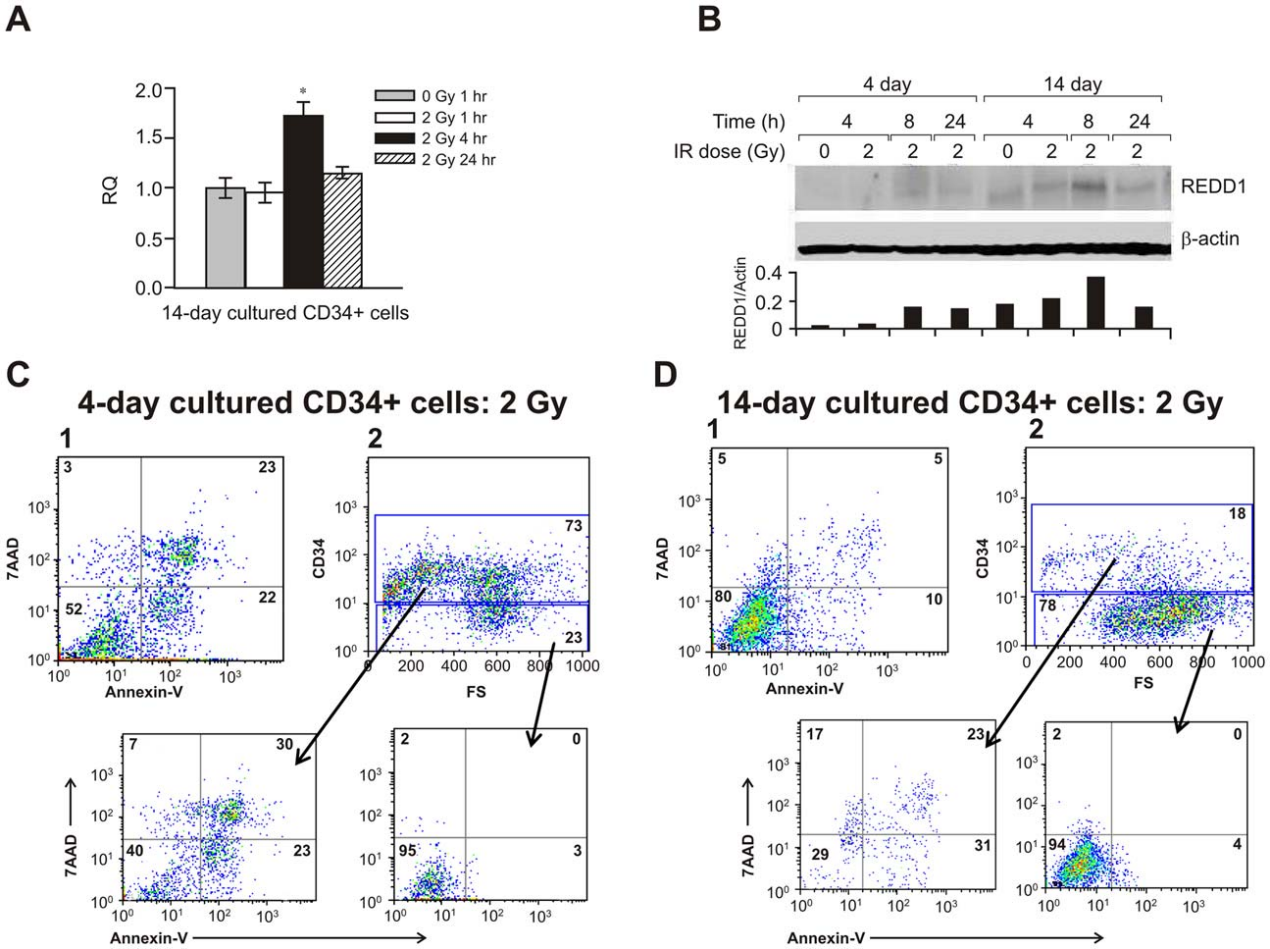
REDD1 expression in human hematopoietic cells. Purified human CD34+ cells were cultured for 4 and 14 days before being exposed to 2 Gy radiation. (A) mRNA levels for *REDD1* in 14-day cultured CD34+ cells using 18S rRNA as a control to calculate the relative quantity (RQ) of gene expression at different times after IR. Means ± SD. *, p <0.05, 2 Gy vs. 0 Gy sham-irradiated control. (B) Western blot shows REDD1 protein levels in cultured CD34+ cells at different times after 2 Gy irradiation. Representative immunoblots and ratio of REDD1/β-actin expression from 3 independent experiments are shown. Flow cytometric analysis for the apoptotic cell death marker Annexin-V/7AAD and CD34 surface marker was performed. Radiation resulted in more apoptotic cells in 4-day (C-1) cultured cells than in 14-day (D-1) culture. (C-2) and (D-2) CD34-positive (progenitor) and CD34-negative (mature) populations were separately gated for Annexin-V/7AAD analysis. FS, forward angle light scatter.

We recently reported that radiation-induced REDD1 expression in human osteoblast cells and REDD1 protected osteoblast cells from radiation damage [4]. In the present study, we tested REDD1 expression in human primary hematopoietic progenitor CD34+ cells before and post-irradiation. Real time RT-PCR and western blot assays confirmed that REDD1 was undetectable in fresh thawed hematopoietic progenitor CD34+ cells regardless of irradiation (data not shown), suggesting that the expression of survival factor REDD1 varied in different cell types after irradiation. Since CD34+ cells are more sensitive to radiation than mature hematopoietic cells [3], we asked whether REDD1 expression in response to radiation was also modulated in specific stages of cell differentiation. To answer this question, CD34+ cells were cultured in serum-free medium with cytokine support as described in Materials and Methods for 4 or 14 days to induce differentiation, associated with loss of the CD34 marker. Following different culture times, cells were exposed to 2 Gy γ-radiations. Half, 1, 4, 8 and 24 h after irradiation, cells were collected and real time quantitative RT-PCR for miR-30c and *REDD1* gene and immunoblotting assays for REDD1 protein expression were performed in samples from both cells cultured for 4 and 14 days. Radiation slightly increased miR-30c expression in both 4 and 14-day cultured CD34+ cells (2 Gy irradiated vs. control, P = 0.07 for 4-day cultures, and P = 0.46 for 14 day cultures, data not shown). Figure 3A showed that *REDD1* gene expression increased by 2 fold in 14 day cultured cells 4 h after irradiation. This result is consistent with our previous finding that *REDD1* gene expression increased at 4 h after irradiation in hFOB cells [4]. REDD1 protein expression revealed a remarkable increase in 14 day cultured cells after 8 h irradiation compared with 4 day cultured cells (Figure 3B).

Next, we evaluated cell apoptosis using flow cytometry and labeling for Annexin-V (apoptotic cell marker) and 7-aminoactinomycin (7AAD, a cell death marker). The viability of cells cultured for 4 and 14 days was >90% before irradiation (data not shown). After irradiation, 7AAD-positive, Annexin-V-positive and double positive apoptotic cells were 48% in the 4-day cultured cells (Figure 3C-1). In contrast, 20% apoptotic cells were detected in the 14-day cultured cells (Figure 3D-1). We further analyzed the frequency of apoptotic cell death in CD34-positive (CD34+) progenitor and CD34-negative (CD34-) differentiated populations from both cultured groups, respectively. There were 73% and only 18% CD34+ cells in 4- and 14-day cultures, respectively (Figure 3C-2 and 3D-2). Interestingly, we found that the majority of apoptosis happened in the CD34+ cell population after irradiation, whereas radiation induced less than 6% apoptosis in differentiated CD34- cells in both 4- and 14-day cultures.

### MicroRNA-30c regulates REDD1 expression in hFOB and 14 day cultured CD34+ cells

**Figure 4:**
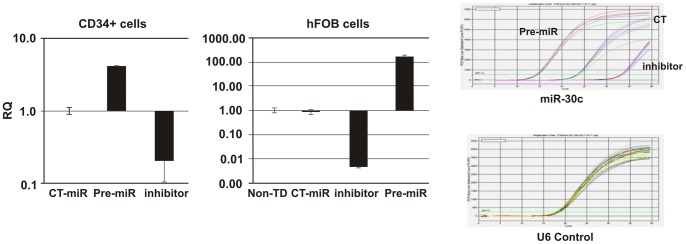
hsa-miR30c regulation by pre-miR30c or miR30c-inhibitor in human CD34+ and hFOB Cells. The pre-miR30c, miR30c inhibitor, or control miR (CT-miR) molecules were transfected into CD34+ cells and hFOB cells. miR30c expression was examined 24 h post-transfection by quantitative RT-PCR. U6 was used as a loading control. RQ: relative quantification. Non-TD: miRNA-untransfected control.

Since miRNA array data showed that miR-30c was decreased in hFOB cells but increased in CD34+ cells after irradiation, we further investigated the effects of miR-30c on REDD1 expression in CD34+ and hFOB cells, using gain and loss of miR-30c expression approaches. The 14 day cultured CD34+ cells or hFOB cells were transfected with miR-30c inhibitor (AM11060) and precursors (pre-miR30c, PM11060) or control-miR from Life Technologies CO. miR-30c expression was examined by quantitative RT-PCR 24 h post-transfection and U6 was used as a loading control. Results shown in Figure 4 demonstrate that transfection of pre-miR-30c enhanced miR-30c expression by 6 fold in 14-day cultured CD34+ cells and 2 logs in hFOB cells. In contrast, transfection of inhibitors suppressed miR-30c expression by 7–8 fold in 14 day cultured CD34+ cells and 2 logs in hFOB cells.

**Figure 5:**
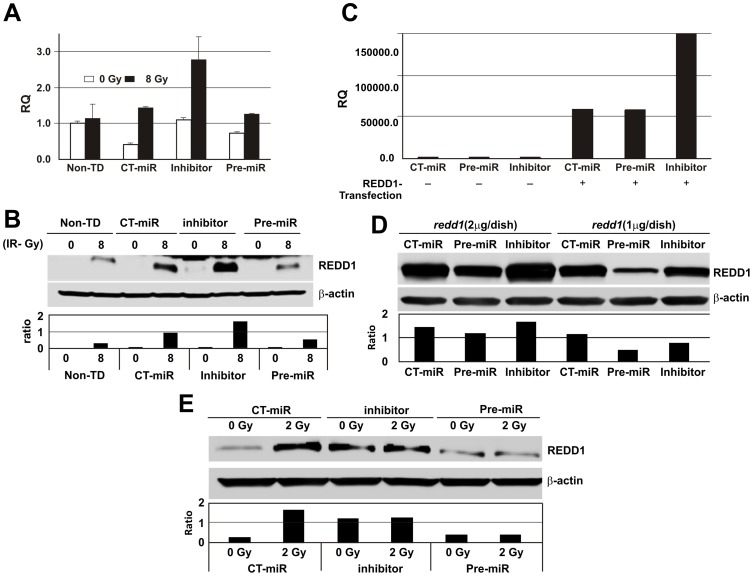
hsa-miR30c regulates REDD1 gene and protein expression in hFOB and CD34+ cells after γ-irradiation. Endogenous REDD1 gene (A) and protein (B) levels were measured after pre-miR30c, miR30c-inhibitor, or control-miR (CT-miR) transfection and γ-irradiation in hFOB cells. Transfection of miR30c-inhibitor upregulated REDD1 gene and protein levels in irradiated samples, whereas radiation-induced REDD1 expression was inhibited by pre-miR30c compared with control-miR transfected sample determined by western blot assays. pre-miR30c, miR30c-inhibitor, or control-miR was co-transfected with either PCMV6-AC-GFP-REDD1 plasmid DNA (1 µg or 2 µg /dish) or vector control. REDD1 gene (C) and protein (D) expression were determined at 24 h post-transfection. Effects of miR30c on REDD1-overexpressing cells are shown. (E) REDD1 protein levels were measured by western blot assays after pre-miR30c, miR30c inhibitor, or control miR (CT-miR) transfection and γ-irradiation in 14 day cultured CD34+ cells (differentiated cells). Radiation-induced REDD1 expression was inhibited by pre-miR30 transfection. Representative expression from 3 independent experiments is shown. Non-TD: miRNA-untransfected control.

Next, the effects of miR-30c on regulation of REDD1expression after γ-irradiation were evaluated in 14 day cultured CD34+ cells and hFOB cells. The cells were exposed to γ-radiation at 24 h after miR30c inhibitor, precursor or miR-control transfection and REDD1 gene and protein expression were measured 24 h after irradiation (48 h post- transfection). Radiation upregulated endogenous REDD1 expression at the gene and protein levels in hFOB cells. Interestingly, transfection of miR-30c inhibitor significantly increased REDD1 mRNA (Figure 5A) and protein (Figure 5B) expression in 8 Gy irradiated hFOB cells, whereas pre-miR30c transfection did not change the gene level of REDD1 but suppressed the radiation-induced REDD1 protein expression in these cells compared with control-miR transfected samples.

To confirm these results, we evaluated effects of miR30c in REDD1-overexpressing hFOB cells. PCMV6-AC-GFP (vector control) or PCMV6-ACGFP-REDD1 plasmid DNA (purchased from OriGENE Bethesda, MD) was co-transfected with miR-30c inhibitor, precursor or control-miRNA molecule into hFOB cells using Lipofectamine 200 reagent. REDD1 mRNA and protein were measured at 24 h after transfection. Overexpression of REDD1 was identified by quantitative RT-PCR (Figure 5C) in REDD1 plasmid DNA- but not vector control DNA transfected-cells. Co-transfection with miR-30c inhibitor enhanced REDD1 gene expression by 2.3 fold compared to co-transfection with control or pre-miR30c. Furthermore, co-transfection of pre-miR30c with different concentrations (1 or 2 µg/dish) of REDD1 plasmid DNA dose-dependently repressed REDD1 protein levels in hFOB cells (Figure 5D). Finally, pre-miR-30c transfection-induced reduction of REDD1 protein levels was also observed in 14 day cultured and 2 Gy-irradiated CD34+ cells (Figure 5E). Together, these results provide direct evidence that REDD1 is a common target of miR-30c.

### MicroRNA-30c regulates survival of osteoblast cells and hematopoietic progenitor cells

**Figure 6:**
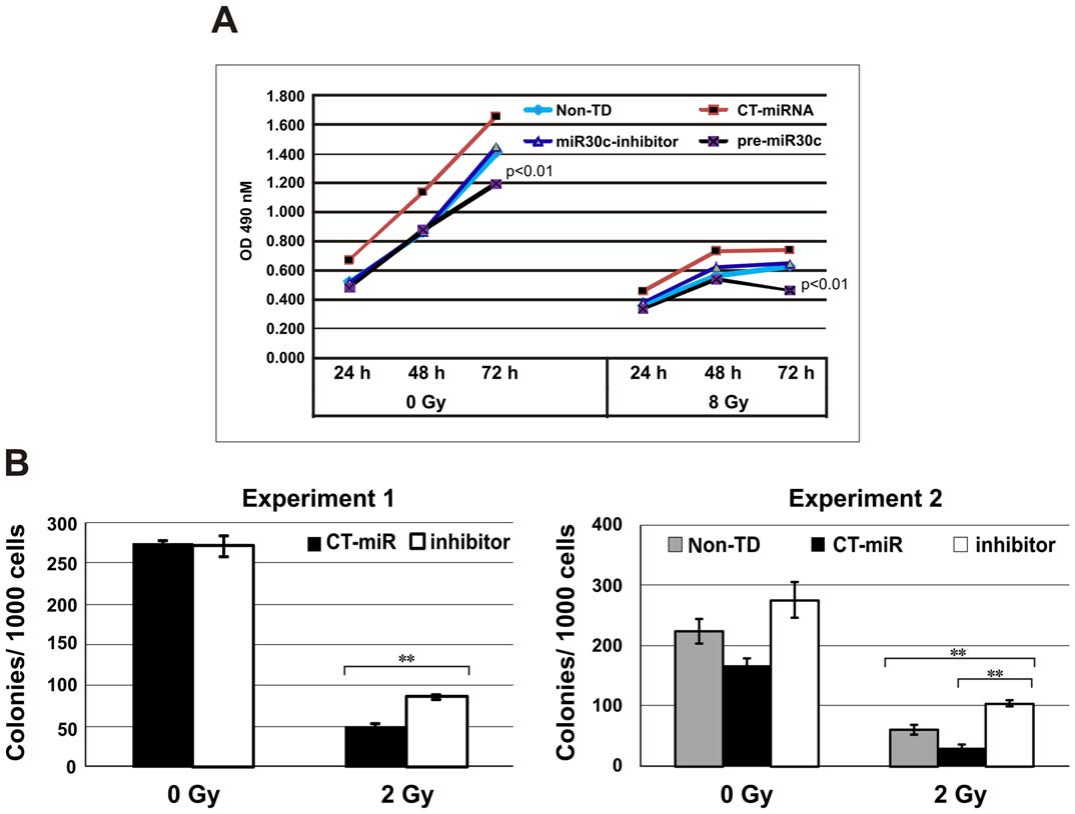
Effects of miR-30c on radiation injury of hFOB and CD34+ cells. (A) Cell death and proliferation potential were measured using the CellTiter 96R AQueous non-radioactive cell proliferation assay (MTS-assay). The quantity of formazan product as measured by the amount of 490 nm absorbance (OD) is directly proportional to the number of living cells in culture. Levels of OD were dramatically lower in pre-miR30-transfected hFOB cells in both irradiated and non-irradiated samples, compared with controls and miR30 inhibitor-transfected samples (p<0.01). Representative expression from 3 independent experiments is shown. (B) Clonogenicity of human hematopoietic progenitor CD34+ cells was quantified in standard semisolid cultures in triplicate. Colonies were counted 14 days later. Results from two experiments showed that transfection of miR30 inhibitor resulted in significant colony number increases in irradiated cells. Means ± SD. **, p <0.01, miR30c inhibitor vs. controls. Non-TD: miRNA-untransfected control.

We asked whether miR-30c expression can influence osteoblast and hematopoietic cell fate since it inhibited REDD1 expression in these cells and our previous study suggested that knockdown of REDD1 resulted in hFOB cell death [4]. After transfection with miR30c inhibitor, precursor or miR-control, hFOB cells were exposed to 8 Gy γ-radiation. hFOB cell survival and proliferation potential were measured using the CellTiter 96R AQueous non-radioactive cell proliferation assay (MTS-assay) at 24, 48 and 72 h post-irradiation. The quantity of formazan product as measured by the amount of absorbance (OD) at 490 nm is directly proportional to the number of living cells in culture. Levels of surviving cells as shown by OD were dramatically low in pre-miR30c-transfected cells in both irradiated and non-irradiated hFOB samples compared with control and miR-30c inhibitor-transfected hFOB cells (Figure 6A; p<0.01), suggesting overexpression of miR-30c induced hFOB cell death.

Finally, we examined the effects of miR-30c on γ-irradiated hematopoietic progenitor CD34+ cells. Since radiation upregulated miR-30s in CD34+ cells, and overexpression of miR-30c resulted in hFOB cell death and inhibition of REDD1 expression, we decided to perform knockdown of miR-30c in CD34+ cells using miR-30c inhibitor. Fresh thawed human hematopoietic progenitor CD34+ cells were transfected with miR-30c inhibitor or control miRNA and were exposed to 2 Gy radiation at 24 h post-transfection of miR-30 inhibitor or control molecule. After irradiation cells were washed and clonogenic assays starting with 1×10^3^ cells/dish were plated in standard semisolid cultures. Colonies were counted 14 days later. Inhibition of miR-30c significantly protected hematopoietic progenitors from γ-irradiation as shown in Figure 6B. Colony efficiencies for unirradiated CD34+ cells ranged from 22–27% in 2 separate experiments, and these efficiencies were not affected by miR-30c-inhibitor. For irradiated cells, colonies in miR-30c inhibitor-transfected cultures were significantly increased compared with control-transfected and untransfected cells. We further examined REDD1 expression in these cells using immunoblotting assay. REDD1 expression was not observed in either miR-30c inhibitor- or control molecule-transfected CD34+ cells (data not shown).

## Discussion

MicroRNAs are post-transcriptional regulators that bind to complementary sequences on target messenger RNA transcripts (mRNAs), usually resulting in translational repression or target degradation and gene silencing [7]. Recent studies suggest roles of miRNAs in responses to physiologic and pathologic stress such as DNA damage in fully developed tissues [9], [19]. DNA damage can transcriptionally induce specific miRNAs and regulate specific cellular functions or influence the expression of a subset of miRNAs by modulating miRNA processing. However, exposure to radiation causes damage to DNA, protein and lipids in mammalian cells, as well as increased mitochondria-dependent generation of reactive oxygen species (ROS), with subsequent cell cycle checkpoint arrest, apoptosis, and stress-related responses [20]. The cellular response to radiation damage is complex and relies on simultaneous activation of a number of signaling networks. No obvious overlap of ionizing radiation-responsive miRNA profiles has been noted among different cells, including primary cells, cancer cells and blood cells [21], [22].Consistent with these reports, in the present study using micro-RNA array we demonstrated that γ-radiation altered the expression of 31 miRNA species (16 downregulated and 15 upregulated) in human hematopoietic progenitor CD34+ cells and 32 miRNA species (14 downregulated and 18 upregulated) in human osteoblast cell line hFOB cells. The profiles of radiation-induced miRNA expression were completely different in CD34+ vs. hFOB cells and these cells have different radiation-toleration phenotypes. Nevertheless, Let-7 and miR-30 families were regulated by radiation in both CD34+ and hFOB cells.

Let-7 was the first known human miRNA and plays a key role in regulation of human cell development and cancer [23]. Dickey et al. [24] reviewed the literature on human miRNA expression in response to radiation and found that the level of Let-7s was significantly altered, either deregulated or upregulated, in different cell types after radiation. Consistent with this, our data show that Let-7f and Let-7g increased in irradiated CD34+ and hFOB cells, respectively. The effects of let-7s on radiation damage in our experimental cell model are under further investigation. Interestingly, miR-30 family members respond to radiation differently in CD34+ and hFOB cells and radiation oppositely regulated miR-30 expression in these cells: miR-30b, miR-30c and miR-30d were upregulated in CD34+ cells whereas miR-30c was downregulated in hFOB cells (Figure 1).

Features of the responses of hematopoietic stem and progenitor cells (HSPC) and hematopoietic niche osteoblast cells to γ-radiation are different. Osteoblasts are relatively more radiation-resistant than HSPCs [18], and the reasons for this are not well understood. We recently reported that the novel cell stress response gene REDD1 is highly induced in mouse bone marrow osteoblastic cells (unpublished data) and human osteoblast cell line hFOB cells after γ-radiation [4]. REDD1 was regulated by p53 and NFkB signaling in response to radiation and plays an important role in suppressing p21-induced cell proliferation arrest and mTOR-induced protein synthesis. These effects of REDD1 were associated with protection of osteoblast cells from radiation-induced premature senescence. In this study, we further demonstrate that REDD1 expression is not observed in fresh thawed hematopoietic progenitor CD34+ cells, however its expression increases with differentiation. The differentiated hematopoietic cells were more radiation-resistant than their precursor CD34+ cells (Figure 3). Using bioinformatics analysis we found a potential binding site of miR-30 in the 3′ UTR of *REDD1* gene. We decided to compare the effects of miR30c in CD34+ and hFOB cells in response to radiation, and asked whether the different features of miR-30c in CD34+ and hFOB cells were associated with their radiation resistance and REDD1 expression.

The effects of miR-30c on REDD1-induced radiation protection in CD34+ and hFOB cells were evaluated using gain and loss of miR-30c function studies. Our data showed that inhibition of miR-30c significantly enhanced endogenous and overexpressed REDD1 gene and protein in hFOB cells. On the contrary, pre-miR-30c repressed REDD1 in these cells. Effects of pre-miR 30c on inhibition of REDD1 were also observed in 14 day cultured CD34+ cells (differentiated hematopoietic cells). Thus, we demonstrate for the first time that REDD1 is a miR-30c target in human hematopoietic cells and their niche osteoblast cells. Furthermore, we addressed the functional activities of miR-30c in survival of radiation-injured CD34+ and hFOB cells. Transfection of miR-30c inhibitor produced a significant increase of clonogenicity in irradiated CD34+ cells and overexpression of miR30c resulted in hFOB cell death. The data suggest that miR-30c plays a key role in radiation-induced cell damage and that this effect may be due, at least in part, to suppression of REDD1 expression. There is abundant evidence of miR-30 in regulation of cell growth, differentiation, apoptosis and senescence in hematopoietic [25], osteoblast [15], adipocyte [26], epithelial and pancreatic cells [27] through different signal transduction pathways. However, effects of miR-30 on ionizing radiation-induced cell damage have not been reported. Our data suggest that CD34+ and hFOB cells have different miRNA expression patterns after irradiation and that radiation-induced miR-30 expression in CD34+ cells may aggravate cell death. Notably, although inhibition of miR30c significantly protected CD34+ cells from radiation damage, it did not induce REDD1 expression in these cells, suggesting that other targets of miR-30c may be involved in radiation-induced stress responses of CD34+ cells. Since multiple potential targets of miRNAs have been suggested (5), validating miR-30 targets in irradiated CD34+ cells will be important to understand the roles of miR-30 in these cells after radiation injury.

There have been a number of studies examining miRNA expression in diseases and identifying specific miRNAs as novel and sensitive biomarkers for disease diagnosis. Radiotherapy is commonly used for cancer treatment and bone marrow toxicity is the dose-limiting factor for radiotherapy in cancer patients. Data from our study suggest that miR-30c plays a key role in radiation-induced hematopoietic cell damage, hence it may be a valuable biomarker of radiosensitivity of hematopoietic cells. This marker could be used to further characterize the toxic radiation doses in individual patients during clinical radiotherapy.

## Materials and Methods

### Cells and γ-irradiation

The human fetal osteoblast cell line (hFOB 1.19) was obtained from the American Type Culture Collection (ATCC, Manassas, VA, USA) and the cells were cultured in a 1∶1 mixture of phenol-free Dulbecco's modified Eagle's medium/Ham's F-12 medium (DMEM-F12, Invitrogen, Carlsbad, CA), supplemented with 10% fetal bovine serum (FBS) (Hyclone, Logan, UT), 2 mM L-glutamine and antibiotics and incubated at 34°C with 5% CO_2_ as described previously [28].

Human primary hematopoietic CD34+ cells were provided by the Fred Hutchinson Cancer Research Center (Seattle, WA). Thawed CD34+ cells were cultured in serum-free medium consisting of Iscove's Modified Dulbecco's Medium (IMDM) supplemented with BIT 9500 (Stem Cell Technologies, Tukwila, WA) and penicillin/streptomycin. Recombinant human (rh) stem cell factor (SCF, 100 ng/ml), rh flt-3 ligand (FL, 100 ng/ml) and rh interleukin-3 (IL-3, 25 ng/ml) were added. All cytokines were purchased from PeproTech, Inc. (Rocky Hill, NJ). The CD34+ cells were incubated at 37°C with 5% CO_2_ [3].

Human hFOB cells and CD34+ cells were γ-irradiated at doses of 0, 2 or 8 Gy (0.6 Gy/min) in the Armed Forces Radiobiology Research Institute Cobalt facility, as described in previous reports [18], [29].

### MicroRNA (miRNA) microarray

Total RNA and miRNA from CD34+ and hFOB cells were extracted using mirVana miRNA isolation kits (Life Technologies) following the manufacturer's protocol. RNA concentrations were determined by measuring OD on the NanoDrop spectrophotometer ND-1000 (Thermo Fisher Scientific) and total RNA quality was verified on the Agilent 2100 bioanalyzer (Agilent Technologies) with RNA 6000 Nano chips.

MicroRNA microarray analysis was performed by LC Sciences (Houston, Texas) on unirradiated or 2 or 8 Gy γ-irradiated human CD34+ and hFOB cells collected 1 h after irradiation. Microarrays utilized µParaflo® microfluidic chip technology and optimized RNA hybridization probes with normalization, enabling highly sensitive and specific direct detection of miRNAs. The Sanger miRBase database (Release 15.0; sanger.ac.uk/Software/Rfam/mirna/) verified microRNA sequences on the array. Multiple control probes were included in each chip. The control probes were used for quality control of chip production, sample labeling and assay conditions. Data were analyzed by LC Sciences using the t-test and ANOVA. The differentially detected ratios (log transformed) of the control and sample signals were generally accepted as true when the p-value was <0.01; these genes were selected for cluster analysis.

### Quantitative real-time PCR

To confirm the microarray results, reverse transcription (RT)-PCR was performed using TaqMan MicroRNA Assays (Applied Biosystems, Foster City, CA) in triplicate according to the manufacturer's instructions. TaqMan miRNA Reverse Transcription Kits were used for RT reactions, and the resulting cDNAs of hsa-miR-30a,-30b, -30c, 30d and 30e were quantitatively amplified on an IQ5 (Bio-Rad) Real-Time PCR System. miRNA levels were normalized to U6 as an internal control.

Quantitative RT-PCR assays for REDD1gene expression were carried out as described [4]. Total RNA was extracted from cells using RNAqueous-4PCR Kits from Ambion (Austin, TX, USA) and was reverse-transcribed using random hexamers. Quantitative real-time PCR was conducted on IQ5 (Bio-Rad) Real-Time PCR System according to the manufacturer's instructions (Bio-Rad, Hercules, CA, USA). Human REDD1 PCR primer and probe (Biosearch Technologies, Inc., 81 Digital Drive, Novato, CA 94949-5728) sequences were as follows:

Forward primer: 5′- GCC AGG TGG GCA AAG AAC -3′.

Reverse primer: 5′- CAC GCT GTG GCA GCT CTT G -3′.

Probe: 5′ Quasar 670-TACTGCGCCTGGCCTACAGC-3′BHQ2.

### Pre-miRNA, miRNA inhibitor and plasmid DNA transfection

Pre-miR30c (PM11060), miR30c-inhibitor (AM11060) or control-miRNA were transfected into hFOB cells using the siPORT NeoFX Transfection Method and CD34+ cells with the Lipofectamine RNAiMAX Method according to the manufacturer's protocol. All reagents and small RNAs were from Life technologies. In brief, the diluted small RNA was mixed with siPORT NeoFX or Lipofectamine RNAiMAX transfection agent and incubated at room temperature, and then dispensed into a culture plate. We then overlaid 10^6^ hFOB or CD34+ cell suspensions onto the transfection complexes and gently tilted the plate to mix. Cells were incubated at 37°C for 24 h.

PCMV6-AC-GFP or PCMV6-AC-GFP-REDD1 plasmid DNA (1 or 2 µg/dish) from OriGene (Rockville, MD) was co-transferred with Pre-miR30c (PM11060), miR30c-inhibitor (AM11060) or control-miRNA into hFOB cells (1.45 million cells per 10 cm dish) using Lipofectamine 2000 reagent (20 µl/dish) according to the manufacturer's protocol (Life technologies). At 24 h after transfection cells were harvested for further analysis.

### Immunoblotting (IB)

One to 5×10^6^ cells from different samples were harvested, washed, and lysed with 1x Laemmli sample buffer. Protein concentrations were determined using a bicinchoninic acid protein assay kit (Pierce). Proteins were separated by sodium dodecyl sulfate polyacrylamide gel electrophoresis and transferred to nitrocellulose membranes. Membranes were preblocked and probed with primary antibodies for REDD1 from ProteinTech (Chicago, IL) and loading controls, according to the manufacturer's instructions, followed by the appropriate horseradish peroxidase-conjugated secondary antibody (Santa Cruz Biotechnology, Inc., Santa Cruz, CA, USA). Signal detection used an enhanced chemiluminescence kit (Thermo Scientific, Rockford, IL, USA) and a Fuji Imaging System.

### Apoptotic flow cytometry and colony-forming assay

Human CD34+ cell expansion and viability (trypan blue-negative cells) from all groups were quantified. Labeling with cell death marker 7-aminoactinomycin D (7AAD) and an apoptotic marker (Annexin-V), and/or CD34 antibody were determined using BD FACSCalibur flow cytometry (BD Biosciences, San Jose, CA). All antibodies and dyes were purchased from BD Biosciences.

Committed hematopoietic progenitors in the CD34+ population were quantitated in standard semisolid cultures in triplicate using 1 ml of Methocult GF+ (Stem Cell Technologies), which consists of 1% methylcellulose in IMDM, 30% fetal bovine serum, 1% bovine serum albumin, 2 mM L-glutamine, 10^−5^ M 2-mercaptoethanol, 50 ng/ml SCF, 20 ng/ml granulocyte macrophage–colony-stimulating factor, 20 ng/ml G-CSF, 20 ng/ml IL-3 and 3 U/ml erythropoietin. Cells from liquid culture were washed twice with IMDM before assays and seeded with 1×10^3^ cells/dish in 35-cm cell culture dishes (from BD Biosciences). Plates were scored for colonies after culturing for 14 days at 37°C, 5% CO_2_ [3].

### Cell proliferation assay (MTS-assay)

MTS [3-(4, 5-dimethylthiazol-2-yl)-5-(3-carboxymethoxyphenyl)-2-(4-sulfophenyl)-2H-tetrazolium, inner salt) assays were performed using the CellTiter 96^R^ AQueous Non-Radioactive Cell Proliferation Assay kit (Promega) according to the manufacturer's protocol. In brief, after irradiation, hFOB cells were plated at 5000 cells/well in 96 well plates in quadruplet. At the indicated times, 20 μl of MTS/PMS solution (ratio 20/1) was prepared and added to the wells containing a final volume of 100 μl medium. The plates were incubated for 4 h at 37°C and the OD at 490 nm was recorded using an ELISA plate reader. The average 490 nm absorbance from three “no cell” control wells was used as blank.

### Statistical analysis

Differences between means were compared by ANOVA and Student's t-tests. *P*<0.05 was considered statistically significant. Results are presented as means ± standard deviations or standard errors of the mean as indicated.
